# Unsupervised classification of neocortical activity patterns in neonatal and pre-juvenile rodents

**DOI:** 10.3389/fncir.2014.00050

**Published:** 2014-05-27

**Authors:** Nicole B. Cichon, Michael Denker, Sonja Grün, Ileana L. Hanganu-Opatz

**Affiliations:** ^1^Developmental Neurophysiology, Neuroanatomy, University Medical Center Hamburg-EppendorfHamburg, Germany; ^2^Institute of Neuroscience and Medicine (INM-6) and Institute for Advanced Simulation (IAS-6), Jülich Research Centre and JARAJülich, Germany; ^3^Theoretical Systems Neurobiology, RWTH Aachen UniversityAachen, Germany; ^4^RIKEN Brain Science InstituteWako-shi, Saitama, Japan

**Keywords:** development, principal component analysis, network oscillations, synchrony, high-frequency oscillations, prefrontal cortex

## Abstract

Flexible communication within the brain, which relies on oscillatory activity, is not confined to adult neuronal networks. Experimental evidence has documented the presence of discontinuous patterns of oscillatory activity already during early development. Their highly variable spatial and time-frequency organization has been related to region specificity. However, it might be equally due to the absence of unitary criteria for classifying the early activity patterns, since they have been mainly characterized by visual inspection. Therefore, robust and unbiased methods for categorizing these discontinuous oscillations are needed for increasingly complex data sets from different labs. Here, we introduce an unsupervised detection and classification algorithm for the discontinuous activity patterns of rodents during early development. For this, in a first step time windows with discontinuous oscillations vs. epochs of network “silence” were identified. In a second step, the major features of detected events were identified and processed by principal component analysis for deciding on their contribution to the classification of different oscillatory patterns. Finally, these patterns were categorized using an unsupervised cluster algorithm. The results were validated on manually characterized neonatal spindle bursts (SB), which ubiquitously entrain neocortical areas of rats and mice, and prelimbic nested gamma spindle bursts (NG). Moreover, the algorithm led to satisfactory results for oscillatory events that, due to increased similarity of their features, were more difficult to classify, e.g., during the pre-juvenile developmental period. Based on a linear classification, the optimal number of features to consider increased with the difficulty of detection. This algorithm allows the comparison of neonatal and pre-juvenile oscillatory patterns in their spatial and temporal organization. It might represent a first step for the unbiased elucidation of activity patterns during development.

## Introduction

Neuronal oscillations are a ubiquitous and robust phenomenon that is observed in various measures of brain activity. As such, they have received much attention as they provide effective means to time the firing of neurons (Fries et al., 2007) and contribute to specific brain states and behavioral abilities (Engel and Fries, 2010; Buzsáki and Wang, 2012). Respecting rather historical than mechanistic reasons, these oscillations have been classified according to their frequency distribution in several bands, among which the most extensively investigated are delta (0.5–4 Hz), theta (4–8 Hz), alpha (8–12 Hz), beta (12–30 Hz), and gamma (>30 Hz) (Buzsaki and Draguhn, 2004). Generally, it is considered that slow oscillations link neuronal networks on a large scale, whereas fast rhythms emerge locally (for review, see Buzsáki and Wang, 2012). The encoding ability of oscillatory activity is increased by functionally relevant frequency sub-bands as well as by temporal coupling of slow and fast rhythms (e.g., theta-gamma code) (for a review, see Lisman and Jensen, 2013).

While the function and major underlying mechanisms of oscillatory activity within adult neuronal networks have been largely investigated and partially elucidated, much less is known about the activity patterns during neocortical maturation. Both human and animal research showed that coupling of neuronal networks in oscillatory rhythms emerges early during brain development (Anderson et al., 1985; Khazipov et al., 2004; Hanganu-Opatz, 2010). The highly discontinuous and fragmented temporal organization as well as the fine scale properties of the activity patterns in immature networks, which remarkably differ from the adult ones (Vanhatalo and Kaila, 2006), have been mainly described after manual detection of events and related to the peculiarity of the recorded neocortical area. For example, in the primary sensory cortices with a columnar organization, spindle-shaped burst oscillations termed as SB represent the dominant activity pattern observed in the local field potential (LFP) and alternate with “silent” inter-burst intervals (Khazipov et al., 2004; Hanganu et al., 2006). They result from intracortical activation, are strongly modulated by other cortical and subcortical areas and seem to act as an early functional template of the later emerging cortical topography (Dupont et al., 2006; Huberman et al., 2006; Hanganu et al., 2007; Yang et al., 2009; Minlebaev et al., 2011). On the other hand, in the association cortices, such as the prelimbic subdivision (PL) of the prefrontal cortex (PFC), the discontinuous oscillations have different properties, SB-like events being accompanied by theta-gamma-coupled oscillations that have been termed as NG (Brockmann et al., 2011). The initial oscillatory entrainment of the PL is driven by hippocampal oscillations in theta frequency band. The directed communication within prefrontal-hippocampal networks during neonatal development seems to be relevant for the ontogeny of recognition memory (Krüger et al., 2012). Despite these prominent differences in their properties, with ongoing maturation, the oscillatory events in all neocortical areas progressively switch to continuous rhythms at juvenile age (Colonnese et al., 2010; Hanganu-Opatz, 2010; Brockmann et al., 2011).

To properly understand the function of these different patterns of early oscillatory activity it is mandatory to objectively characterize and distinguish them. This can be optimally achieved by using automatic analysis algorithms, as shown for human data (Vanhatalo et al., 2005; Chipaux et al., 2013). Unfortunately, this task is very difficult when the detection and characterization of the events is performed manually, as it has been done in the past for animal data (Khazipov et al., 2004; Sitdikova et al., 2014). The reported activity patterns in the neonatal cortices and their properties appear highly diverse, even when the same area and age were investigated. This heterogeneity is even more obvious for the oscillations during the transition period from discontinuous to continuous activity during the pre-juvenile age. To decide whether the reported heterogeneity is a genuine feature of developing networks it is mandatory to exclude other possible causes, such as analytical or acquisition system-dependent differences. In this line, development of an unsupervised method for unbiased categorization of activity patterns in the developing brain would be an important step. It would equally enable their further characterization, such as the assessment of the spatial and temporal organization over a specific cortical area during development.

In this work, we describe a novel unsupervised method for detecting and classifying the different patterns of oscillatory activity in developing neocortices of anesthetized rodents. This method considered the root mean square (rms) of the recorded signal for the detection of early oscillatory events. The major features exhibited by these events were used for their classification based on a principle component analysis (PCA) and a fuzzy clustering algorithm. We calibrated the method for its reliability and yield on the prelimbic SB and NG from neonatal rats, the developmental stage at which these events are most clearly distinguishable. Therefore, we analyzed to which extent even single features may allow a robust classification. In the Results section we highlighted two applications of our method. First, we showed that the method performed equally well in classifying the neonatal activity in the primary visual cortex (V1) of the rat and the PFC of the mouse. With the developmental switch from discontinuous to continuous network oscillations in pre-juvenile rats (Brockmann et al., 2011) the categorization of SB and NG becomes difficult to perform manually as the individual features describing the events are less well separated with increasing age. For such data, a combination of multiple features of events led to optimal classification. Second, we demonstrated how the classified oscillatory events can be further processed, for example in synchrony analysis to uncover the specific spatial and temporal organization of the network entrainment during development.

## Materials and methods

In the first part of this section we described the experimental paradigms and general analytical tools. In the second part we developed and calibrated a new three step unsupervised method for event classification. For this, we firstly showed how to initially detect discontinuous oscillatory events. We focused on the two major patterns of discontinuous oscillatory activity in the neonatal PL, the SB and NG. Subsequently, we quantified 11 features based on single oscillatory events, which captured the main characteristics of SB and NG. Finally, we described how to automatically cluster events into SB and NG oscillations based on a vector composed of these features. We concluded this section with a validation of the method by quantifying its classification performance by comparison to a manual classification of events in data from neonatal rats.

### Surgical preparation and recording protocols

All experiments were performed in compliance with the German laws and the guidelines of the European Community for the use of animals in research and were approved (proposal number 94/08 and 111/12) by the local ethical committee. Pregnant Wistar rats were obtained at 14–17 days of gestation from the animal facility of the University Medical Center Hamburg-Eppendorf, housed individually in breeding cages with a 12 h light/12 h dark cycle and fed *ad libitum*.

**Figure 1:**
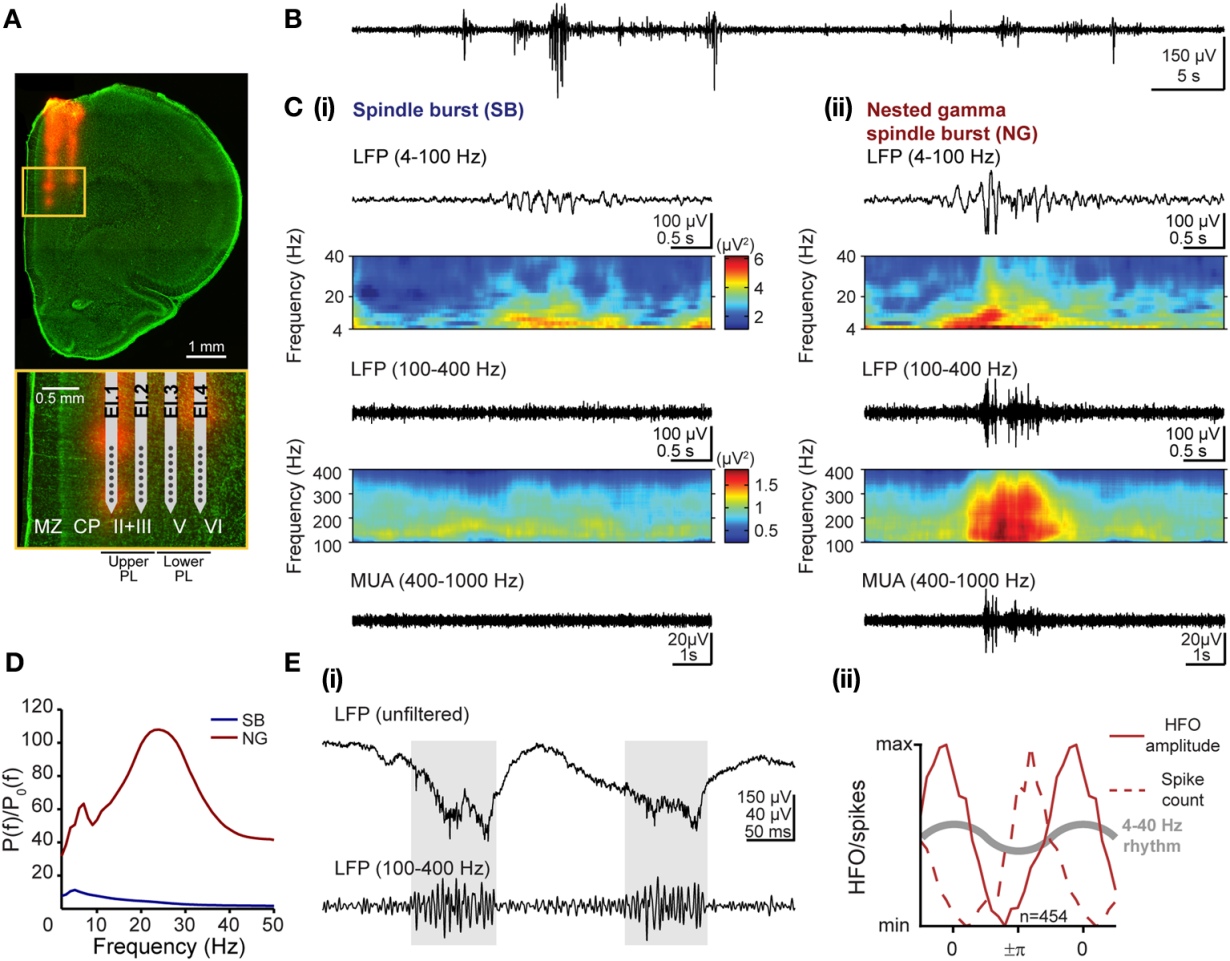
**Patterns of network activity and their frequency components in the developing prelimbic cortex of rats. (A)** Digital photomontage reconstructing the location of the DiI-labeled 4-shank recording electrode (orange) in the PFC of a Nissl-stained 100 μm-thick coronal section (green) from a P8 rat. Inset, drawing of the 4 × 8 electrode with horizontal spacing of 200 μm and vertical spacing of 50 μm according to the DiI-stained tracks within upper and lower cortical layers, which are displayed at higher magnification (yellow box). **(B)** Extracellular LFP recording (4–100 Hz) of the discontinuous oscillatory activity in the PFC of a P8 rat. **(C)** Examples of characteristic SB **(i)** and NG **(ii)** displayed after band-pass (top: 4–100 Hz, bottom: 100–400 Hz) filtering and accompanied by the color-coded frequency plots showing the wavelet spectra of the LFP at identical time scales. Both plots for one filter range are scaled identically corresponding to the color bars shown in **(i)**. **(D)** Averaged power spectra *P(f)* for SB (blue) and NG (red) during the neonatal developmental stage and normalized by the baseline power *P*_0_(*f*) of time windows that did not exhibit an oscillatory SB or NG event. Note the common frequency component in theta-alpha frequency band for both prelimbic patterns of oscillatory activity and the additional beta-low gamma component that is most prominent in NG. **(E)** Distinct features of high-frequency oscillations and spiking activity. **(i)** Extracellular LFP recording in upper PL of two theta cycles superimposed with nested HFOs (marked by gray background) displayed before (top) and after filtering (100–400 Hz, bottom). Note that HFOs are visible in the unfiltered signal. **(ii)** Normalized histograms of HFO amplitude (solid lines) and spiking probability (dotted lines) as a function of theta-alpha phase in the neonatal lower PL of 5 rats. The reference theta-alpha cycle for NG is sketched as gray curve.

Extracellular recordings were performed from the PFC (1.5–2.5 mm anterior to bregma suture, 0.1–1 mm from the midline) and V1 (0.5–1 mm anterior to lambda suture and 2–3 mm from the midline) of postnatal day (P) 7–12 male rat pups as well as from the PFC (0.5–0.7 mm anterior to bregma suture and 0.3 mm from the midline) of P9-10 mice using experimental protocols previously described (Brockmann et al., 2011). Under light urethane-anesthesia (1 g/kg; Sigma-Aldrich, Taufkirchen, Germany) that mimics the full spectrum of natural sleep (Clement et al., 2008) and had no major effect on the neonatal network activity (Yang et al., 2009), the head of the pup was fixed into the stereotaxic apparatus (Stoelting, Wood Dale, IL) using two metal bars fixed with dental cement on the nasal and occipital bones, respectively. The bone over the PFC was carefully removed by drilling holes of less than 0.5–1 mm in diameter. Removal of the underlying dura mater by drilling was avoided, since leakage of cerebrospinal fluid or blood damps the cortical activity and spiking activity (Hanganu-Opatz, personal observations). The body of the animals was surrounded by cotton and kept at a constant temperature of 37°C by placing it on a heating blanket. After a 20–40 min recovery period, multielectrode arrays (Silicon Michigan probes, NeuroNexus Technologies, Ann Arbor, MI) were inserted into the PFC perpendicularly to the skull surface and parallel to the midline until a depth of 1.5–3 mm. The electrodes were labeled with DiI (1,1'-dioctadecyl-3,3,3',3'-tetramethyl indocarbocyanine, Invitrogen, Darmstadt, Germany) to enable post-mortem the reconstruction of electrode tracks in histological sections of the PFC (Figure 1A). One silver wire was inserted into the cerebellum and served as ground and reference electrode. Miniature earphones placed under the pup's body were sensitive enough to detect the smallest visible movements of the limbs as well as the breathing of pups during recordings.

Simultaneous recordings of the LFP and multi-unit activity (MUA) were performed from the PFC using four-shank 32-channel Michigan electrodes (0.5–3 MΩ). The eight recording sites of each shank were separated by either 50 or 100 μm, while shanks were separated by 200 μm. The recording sites of multiple shanks covered the entire depth of the prefrontal sub-division PL (Van Eden and Uylings, 1985). Both LFP and MUA were recorded for 40–60 min at a sampling rate of 32 kHz using a multi-channel extracellular amplifier (Digital Lynx 4S, Neuralynx, Bozeman, MO) and the corresponding acquisition software (Cheetah). During recording the signal was band-pass filtered between 0.1 and 5 kHz.

The data were split into two age groups containing the recordings from neonatal (P7-9) and pre-juvenile (P10–12) pups, respectively, (*n* = 6 for both groups). The data from the PL of neonatal rats were used for the development and calibration of the method, as highlighted in this section, whereas the data from the neonatal rat V1 and mouse PL as well as from the PL of pre-juvenile rats were used for assessing the performance of the developed method during the transition period from discontinuous to continuous activity (shown in the Results section).

### Histology and immunohistochemistry

Neonatal and pre-juvenile rats were deeply anesthetized with 10% ketamine (aniMedica, Senden-Bösensell, Germany)/2% xylazine (WDT, Garbsen, Germany) in NaCl (10 μ l/g body weight, i.p.) and perfused transcardially with 4% paraformaldehyde dissolved in 0.1 M phosphate buffer, pH 7.4. The brains were removed and postfixed in the same solution for 24 h. Subsequently, coronal slices were sectioned in the coronal plane at 100 μm and stored at −80°C.

For the reconstruction of DiI-labeled electrode tracks into the PFC, fluorescent Nissl staining was performed as previously described (Quinn et al., 1995) using the NeuroTrace® 500/525 green fluorescent Nissl stain (Invitrogen). Briefly, rehydrated slices were incubated for 20 min with 1:100 diluted NeuroTrace. Sections were washed, coverslipped with Fluoromont and examined using the 488 and 568 nm excitation filter of the Imager M1 microscope (Zeiss, Oberkochen, Germany). All photographs were adjusted for brightness and contrast with Adobe Photoshop CS4.

### Data analysis

Data were imported and analyzed off-line using custom-written tools in Matlab software version 7.7 (Mathworks, Natick, MA). For the analysis of LFPs, the signals were low-pass filtered (<1500 Hz) using a third order Butterworth filter before reducing the sampling rate to 3255 Hz. All filtering procedures were performed in a manner preserving phase information. As previously demonstrated (Brockmann et al., 2011), little, if any, passive propagation of LFP signals has been detected. To extract the spiking activity, the raw electrode signal was firstly high-pass filtered (>407 Hz) and a threshold for the detection of spike waveform was individually set depending on the geometry of the recording site. The stored signals were sorted into similar waveform shapes using the Offline Sorter Software (Plexon, Dallas, TX). Since assessment of the individual recording sites to the same cortical layers was not possible over the neonatal and pre-juvenile development, we distinguished the activity in the upper and lower PL by separately analyzing data from the recording sites of the most medial two shanks (El.1, El.2) and most lateral two shanks (El.3, El.4), respectively (Figure 1A).

Data in the text are presented as the mean ± standard deviation (*SD*) and displayed as bar diagrams. Comparisons of groups were performed using the non-parametric Kolmogorov-Smirnov test. Significance levels of *p* < 0.05 (^*^), *p* < 0.01 (^**^), or *p* < 0.001 (^***^) were used.

#### Average power spectra of oscillatory events

The short duration and variable frequency of oscillatory events covering multiple bands (from theta to gamma) required noise reduction in calculating the power spectra averaged across oscillatory events. To this end, we determined the power content in band-pass filtered versions of the recorded signal (Pfurtscheller and Lopes da Silva, 1999). The band-pass filters were centered on frequencies *f* from 1 to 50 Hz and their bandwidth was defined as function of the period (*T* = 1/*f*) of the oscillation. Lower and upper cut-off frequencies were set to (1.2 *T*)^−1^ and (0.8 *T*)^−1^, respectively. To improve the accuracy of calculation in narrow filter bands, a FIR filter (eegfilt function from the EEGLAB toolbox, Delorme and Makeig, 2004) was used. For all detected oscillatory events the total power *P(f)* of a signal filtered at center frequency *f* was calculated and averaged across events of the same type. Similarly, the average baseline power spectrum *P*_0_(*f*) for all epochs without oscillatory activity was determined. Finally, the normalized power spectra were then defined as *P*(*f*)/*P*_0_(*f*).

#### Coherence

As spectral measure of correlation between two signals coherence was calculated from the cross-spectral density between the two signals and normalized by the individual power spectral density of each (Jerbi et al., 2007). The computation was performed using a multi-taper approach implemented in the chronux toolbox (chronux.org^1^, Mitra and Bokil, 2008) according to the formula

$$C(f)=\frac{\overset{N}{\underset{i=1}{\sum }}X_{i}(f)Y_{i}^{*}(f)}{\sqrt{\overset{N}{\underset{i=1}{\sum }}|}X_{i}(f){|}^{2}\sum_{i=1}^{N}|Y_{i}(f){|}^{2}}$$

where *X*_*i*_(*f*) and *Y*_*i*_(*f*) are the Fourier transforms of the respective two signals for the *i-*th taper (*N* = 5) at frequency *f*, and ^*^ indicates the complex conjugate. The coherence coefficient is given as the modulus of the complex-valued coherence *C(f)*.

Coherence coefficients were assessed for all pair-wise combinations of the LFP from one reference recording site (the deepest site on the shank closest to the midline, which was marked by X in Figures 8Aii,Bii) and all 31 remaining recording sites spanning the entire depth of the PL. The computation was performed on a time series that was constructed by concatenating all time periods including detected oscillatory events. The coherence coefficients of individual oscillatory episodes were separately averaged for the frequencies in the 4–12 Hz (theta/alpha) and in the 16–40 Hz (beta/low gamma) frequency bands.

### Unsupervised method for the identification and classification of patterns of discontinuous oscillatory activity

As we previously reported, the patterns of population activity in the PL, as reflected in the LFP, critically depend on age (Brockmann et al., 2011). During the first postnatal week discontinuous network oscillations develop in the prelimbic subdivision of the PFC (Figure 1B) as a result of directed communication with the hippocampal CA1 area. Only during the second postnatal week, this discontinuous activity gradually switches to continuous activity that emerges through mutual interactions within prefrontal-hippocampal networks. Correspondingly, the discontinuity index calculated as the fraction of time lacking network activity from the total recording time progressively decreased from 0.77 ± 0.1 for P8 rats (*n* = 4) to 0.65 ± 0.25 for P12 rats (*n* = 3).

#### Detection of discontinuous patterns of oscillatory activity

In a first step, we aimed to automatically identify the discontinuous oscillatory events using a threshold-based detection. According to our previous data (Brockmann et al., 2011), the occurrence of discontinuous oscillations increased with age, while their amplitude decreased. Therefore, the threshold for automatic detection of discontinuous events must be set according to the signal-to-noise ratio of each recording, but independently of the absolute amplitude and number of oscillations.

Here, we detected discontinuous oscillations in the neonatal PL as deflections of the rms of the band-pass (4–100 Hz) filtered signal (calculated in a sliding window of 200 ms) that exceeded a recording-specific threshold. To determine this threshold, a histogram of the rms was constructed from a 5 min-long time segment of each data set, which started 15 min after the onset of the recording (to exclude any type of boundary effect) and was confirmed by visual inspection to contain oscillatory events (Figure S1). The bulk of lowest rms values were identified by fitting a Gaussian function $f\left(x\right)=ae^{-\frac{{\left(x-\mu \right)}^{2}}{2\sigma^{2}}}$ to the histogram entries corresponding to rms values ranging from zero up to the largest peak of the histogram, where the fit parameter μ represented the mean and σ denoted the SD of the Gaussian distribution. Based on this fit, time windows containing rms values ≥μ + 2σ corresponded to oscillatory events, whereas time windows containing rms values <μ + 2σ were considered as epochs of network “silence” (i.e., inter-burst event intervals of low signal power). Subsequently, fragmented detection of events was avoided by identifying all consecutive oscillations with inter-event intervals <100 ms as a single event (i.e., including the time between two events). Only oscillations lasting >1 s were considered for further analysis.

#### Characterization of SB and NG

Visual inspection of these discontinuous events led to the classification into SB and NG (Brockmann et al., 2011; see Figures 1Ci,ii for two representative examples). The qualitative differences in the time series of SB and NG events were also reflected in their spectral composition. In Figure 1C, the corresponding time-frequency plots of the LFP were obtained by Morlet wavelet analysis for frequencies ranging from 4 to 100 Hz as well as from 100 to 400 Hz. Due to high differences in absolute power the analysis was performed separately for both frequency ranges to facilitate visualization.

The complexity and variability of the frequency and amplitude distributions observed across multiple discontinuous patterns of prelimbic activity requires long-lasting expertise for their characterization and classification by visual inspection. Consequently, the procedure of categorizing events as SB or NG might be biased by the researcher performing the analysis. Likewise, the high degree of variability between SB and NG makes them difficult to be described in a quantitative fashion. In the following, we therefore aimed at better characterizing these two types of events that constitute the discontinuous activity in the PL, in order to understand, which characteristics may be suitable for their differentiation.

We quantitatively assessed the characteristics that define the SB and NG extracellularly recorded in the PL of neonatal (P7–9) urethane-anesthetized rats (*n* = 6) *in vivo.* Periods with oscillatory events were visually classified into SB and NG and separately analyzed. While of short duration (SB: 1.94 ± 0.26 s; NG: 2.87 ± 0.61 s), the prelimbic discontinuous events had a highly variable frequency distribution across episodes, and oscillation frequencies may rapidly switch within one event from slow theta/alpha to fast beta/low-gamma bands and vice versa (Figure 1C). To identify the dominant frequencies and to decide whether they differ between SB and NG, we calculated the average power spectra across individual oscillations relative to baseline, defined as the activity between oscillatory events. For neonatal rats, the resulting normalized power spectra revealed that SB had a dominant frequency within the theta band (5 Hz), whereas NG showed larger power and two distinct frequency peaks confined to theta (7 Hz) and beta/low-gamma (24 Hz) bands (Figure 1D). As suggested by the visual inspection of the traces, these differences originated from the fast oscillatory cycles of large amplitude which were interspersed within the slower theta/alpha band rhythm in NG, but rarely in SB.

Besides these slow oscillatory components, short episodes of fast low-amplitude oscillations superimposed the NG as nested events (Figure 1Cii). Their high frequency ranging from 100 to 400 Hz led to the question whether they represent oscillatory events or “spectral leakage” of the extracellular spike shapes into several frequency bands (Jackson et al., 2011; Ray and Maunsell, 2011). To answer this question, we tested our data for two previously reported differences between high-frequency oscillations (HFOs) and spike-leaked high-frequency oscillations (SLHFOs) (Scheffer-Teixeira et al., 2013; Tort et al., 2013). First, identification of HFOs, but not SLHFOs can be achieved by inspection of the unfiltered signal. For our data, nested fast oscillations were clearly visible not only in the wavelet spectra but also in the unfiltered LFP trace (Figure 1Ei). Second, for SLHFOs the preferred phase at which the high frequency activity couples to the theta cycle coincides with the preferred theta phase of spiking. To decide whether the instantaneous phase of the slow LFP oscillation with frequency *f*_*p*_ modulated the amplitude of the fast oscillation with frequency *f*_*a*_, the Hilbert transform was used to calculate the analytic signals of the band-pass filtered LFPs at low (4–40 Hz) and high (100–400 Hz) frequencies (Le Van Quyen et al., 2001). We estimated the time series of the instantaneous phase of underlying oscillations in NG (ϕ_*f*_*p*__(*t*)) by taking the argument of the slow (4–40 Hz) analytic signal, whereas by taking the modulus of the fast (100–400 Hz) signal we estimated its amplitude (*A*_*f*_*A*__(*t*)). The resulting amplitude *A*_*f*_*A*__(*t*) was described as a function of ϕ_*f*_*p*__(*t*) by binning the phase axis with a bin size of π/10 and plotting the average amplitude corresponding to phases that fall in a given bin. We contrasted the graph to the histogram of the total number of spikes found at a specific phase ϕ_*f*_*p*__(*t*) of the LFP. We observed that the preferred phase of the slow signal component at which nested fast oscillations were detected varied with respect to the preferred spike-triggered phase of the slow component. In particular, in the lower PFC, the amplitude of the high frequency activity peaked at a later phase than the preferred phase of spikes within the 4–40 Hz oscillatory cycle (Figure 1Eii), suggesting that they are not of the same origin (phase of 0 refers to the peak, phase of +π/−π refers to the trough of the cycle). Thus, our results based on these two criteria indicated that the episodes of nested fast oscillations superimposed on NG do not represent SLHFOs, but HFOs.

#### Quantification of distinct features for single SB and NG

**Figure 2:**
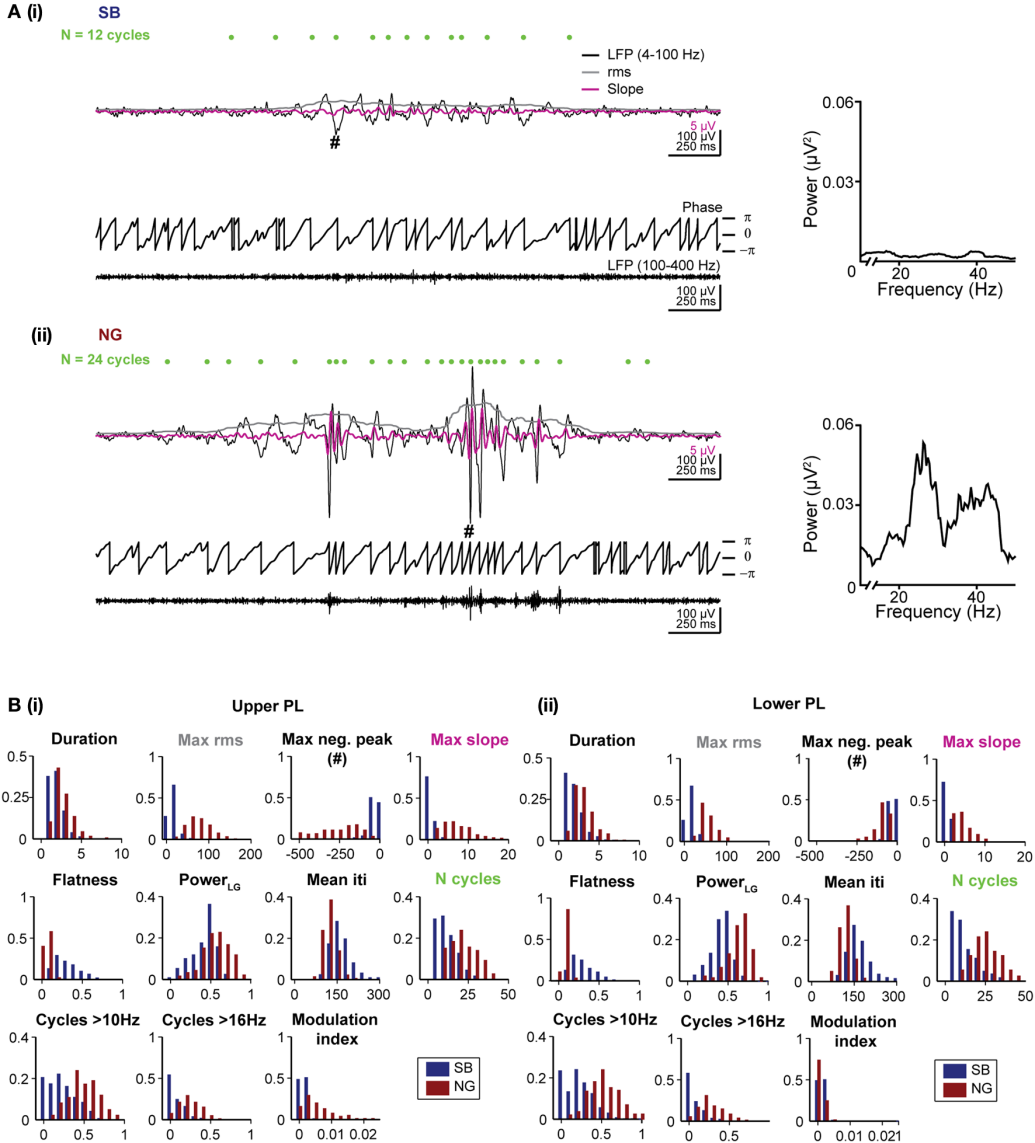
**Distinct features of the SB and NG in the PL of neonatal rats. (A)** Top, characteristic SB **(i)** and NG **(ii)** displayed after band-pass filtering (4–100 Hz) accompanied by the rms amplitude (gray trace) and the slope of the signal (magenta trace). Green dots mark the detected troughs of the signal. Bottom, the phase of the filtered (4–100 Hz) signal and the signal after band-pass filtering between 100 and 400 Hz. The hash symbol marks the maximum negative peak of the event. Right, the corresponding power spectra for frequencies between 10 and 40 Hz. **(B)** Histograms displaying the distributions of values for each of the eleven distinct features of SB (blue bars) and NG (red bars) in the upper **(i)** and lower **(ii)** PL.

The analysis above revealed that on average the quantitative differences between SB and NG arise (i) from their different durations and amplitudes, (ii) from additional beta/low-gamma cycles with high amplitude present only in NG events, and (iii) the presence of nested fast oscillations (i.e., HFOs) during NG events. In the following, we identified 11 features solely based on single oscillatory events that capture these properties. As reference, the features were visualized in examples shown in Figures 2Ai,ii.

Duration and amplitude were quantified by five features. The event *duration* was trivially quantified as the duration of the automatically detected event. Differences in the amplitude of events were captured by four features. The values for the *maximum rms* and the largest negative deflection, *maximum negative peak*, are expected to differ strongly for SB vs. NG as they directly measure the event amplitude. In addition, the *maximum slope* of the signal filtered between 4 and 40 Hz was chosen as a sensitive measure for the steep increase associated with beta-low gamma cycles during NG. Finally, we measured the *flatness* of each oscillatory event defined as the minimum rms value divided by the maximum rms value of the event, which yields a measure of the amplitude in relation to its baseline. All rms values were calculated as described above for the event detection. The sliding window of 200 ms used to calculate the rms was sufficiently large to capture at least one cycle of the slow oscillatory rhythm.

The spectral composition of SB and NG was also quantified by a total of five features. First, the total raw power in the beta/low gamma band was calculated as the power content between 16 and 40 Hz normalized by the total power between 4 and 50 Hz (indicated as *Power LG*). Since power spectra of individual oscillatory episodes were typically noisy due to the short duration of the events, potential differences in the duration of cycles of the event or their regularity as measured by the mean time between two consecutive negative peaks (inter trough interval, iti) were considered (*mean iti*). We additionally counted the total number of cycles (indicated as *N cycles*), which is expected to be larger for NG than SB due to the longer duration of NG and their increase in the number of beta/low gamma cycles. In addition, the number of cycles with a duration corresponding to more than 10 Hz (*N cycles > 10 Hz*), and to more than 16 Hz (*N cycles > 16 Hz*) were used as features aimed to directly assess differences in the number of beta/low gamma cycles. To identify oscillatory cycles in these measures, we detected positive and negative peaks with a time lag of at least 25 ms between consecutive peaks (i.e., frequency 40 Hz) and a difference in amplitude between two consecutive positive and negative peaks of at least 2 *SD* of the noise amplitude.

As a final feature, we quantified the presence of nested HFO by evaluating the cross-frequency coupling by using the *modulation index* (Tort et al., 2010). To this end, for each event ϕ_*f*_*p*__(*t*) and *A*_*f*_*A*__(*t*) were calculated as described above. The phase axis was then binned and for each bin the mean amplitude was calculated (≤〈*A*_*f*_*A*__〉_ϕ_*f*_*p*___(*j*): *A*_*f*_*A*__, averaged over all time points where ϕ_*f*_*p*__(*t*) falls in the j-th phase bin) and normalized by the sum over all bins

$$P\left(j\right)=\frac{{\langle A_{f_{A}}\rangle }_{\phi_{f_{p}}}(j)}{\sum_{k\;=\;1}^{N}{\langle A_{f_{A}}\rangle }_{\phi_{f_{p}}}(k)}$$

where *N* = 20 is the number of phase bins (bin size: π /10). Phase-amplitude coupling is detected if the amplitude distribution *P(j)* deviates from the uniform distribution *Q*. The deviation was quantified using the Kullback–Leibler (KL) divergence:

$$D_{KL}\left(P,\;Q\right)=\sum_{j\;=\;1}^{N}P\left(j\right)\log\left[\frac{P\left(j\right)}{Q\left(j\right)}\right]$$

The modulation index is obtained by normalizing the KL divergence to 1:

$$MI=\frac{D_{KL}\left(P,\;Q\right)}{\log\left(N\right)}$$

A uniform phase–amplitude distribution (*MI* = 0) indicates that the amplitude of the frequency component *f*_*a*_ is independent of the phase of the oscillation at *f*_*p*_. In contrast, *MI* = 1 reflects perfect phase–amplitude coupling between superimposed fast oscillations and the underlying NG [within the precision prescribed by the binning of *P(j)*].

In summary, we calculated the following features: (1) duration of events, (2) maximum rms, (3) maximum negative peak, (4) maximum slope, (5) flatness, (6) beta/low gamma power, (7) mean iti, (8) N cycles, (9) N cycles > 10 Hz, (10) N cycles > 16 Hz, (11) modulation index. In Figures 2Bi,ii we showed the distribution of these 11 features for manually classified SB and NG in the upper and lower PL, respectively. While all features exhibited significant differences (*p* < 0.001) in the respective distributions of SB and NG, the distributions nevertheless overlap to varying degrees and consequently, none of the features allowed to trivially differentiate the events, e.g., using a hard threshold. Moreover, this would have required individual fine tuning of the thresholds with respect to the distributions obtained for the upper and lower layers of the PL and for different recording sessions. Therefore, a manual classification based on imposing a threshold on a single feature was rather difficult.

#### Automatic feature-based classification of SB and NG

We introduced an unsupervised classification method that distinguished SB and NG based on the combination of features. A feature vector for each oscillatory event was assembled by concatenation of its features. All resulting vectors were processed by principal component analysis (PCA) and the dimensionality of data was reduced to the first *K* principal components (PCs). For each oscillatory event the probability of being either an SB or an NG was assessed by using the Gustafson–Kessel clustering algorithm (Babuska et al., 2002) with an implementation of the Fuzzy Clustering and Data Analysis Toolbox (Abonyi and Feil, 2007). Fixing the number of clusters to two, the algorithm assigns to each oscillation membership degrees (numbers between 0 and 1) with respect to the two clusters corresponding to SB and NG (the sum of membership degrees is 1). The method is an extension of the k-means algorithm and is based on minimizing the summed weighted variances between all combinations of feature vectors and cluster centers. The optimal weights correlate with the corresponding membership degrees. The employed algorithm is able to handle data clusters with different shapes by additionally allowing for adaptive and cluster-specific vector norms, which are simultaneously determined in the optimization procedure. By putting a threshold on the membership degrees, it was possible to group oscillations as SB, if the membership degree to the SB cluster exceeded this threshold, as NG, if the membership degree to the NG cluster exceeded this threshold, and as unclassified (UC), if both membership degrees are below the threshold. In the following, only oscillations with a membership degree exceeding a threshold of 0.7 for one of the two clusters were considered for further analysis and correspondingly assigned as SB or NG. Lowering or raising the threshold changed the sensitivity of the analysis.

The used fuzzy clustering approach was of practical advantage by providing a direct mechanism to control the number of events that remained UC when varying the threshold. However, the choice of clustering is not essential to the approach, since comparable classification could be obtained using standard unsupervised classifiers such as k-means clustering, however without identifying UC events (see Discussion).

#### Validation of the unsupervised classification method

**Figure 3:**
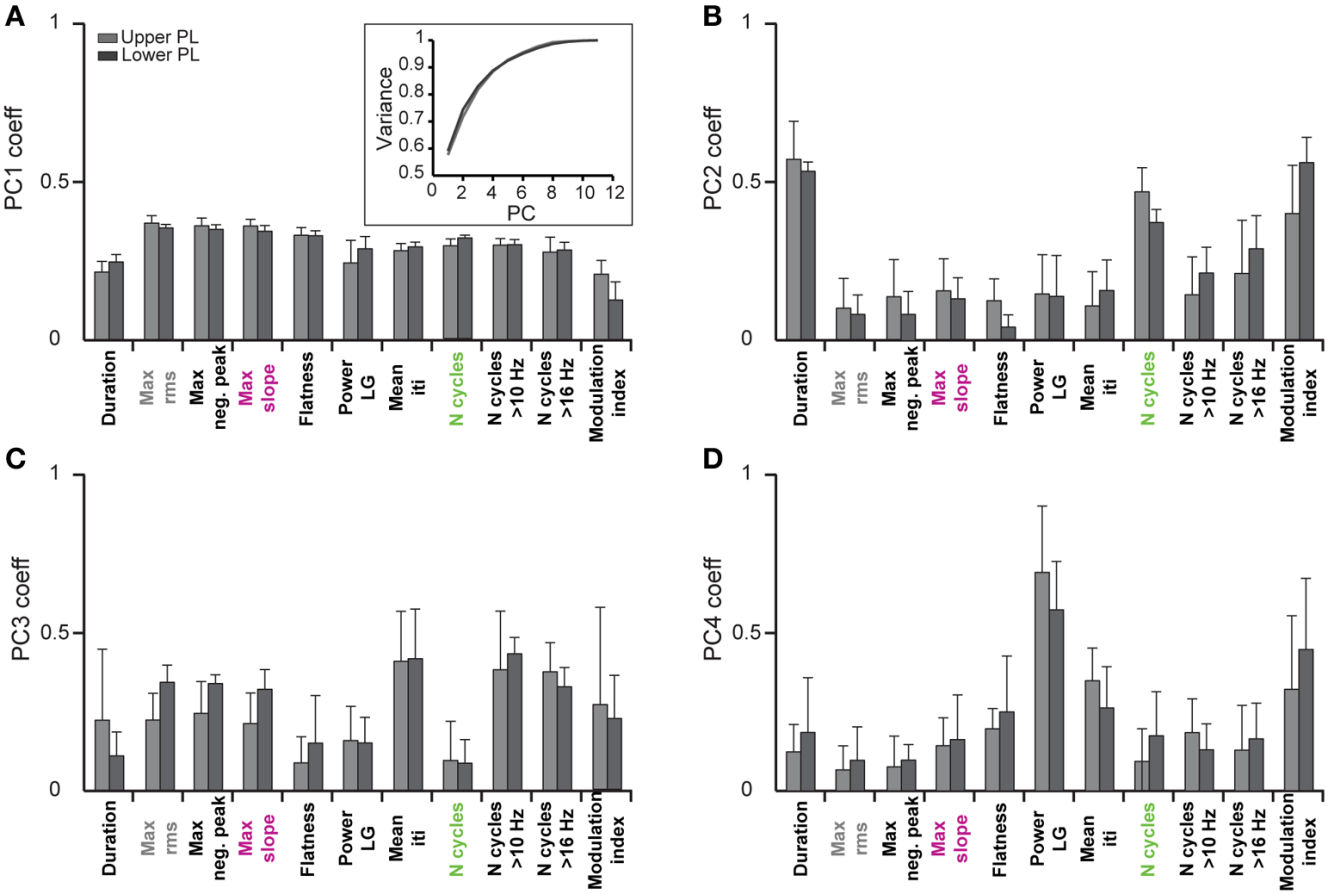
**Contribution of single features to the clustering of oscillatory events by principal component analysis**. Bar diagrams show the average PCA coefficients (*n* = 6 pups) that relate the contribution of each of the eleven features to the first four principle components (PC1–4) in **(A–D)**, respectively. Inset, cumulative variance covered by the principal components.

In a first step we investigated which features contribute to the individual PCs. All subsequent analyses were separately performed by pooling data from the upper two or lower two shanks corresponding to the upper and lower PL, respectively, to account for possible layer-specific differences in the characteristics of oscillatory events (Figure 2B). In both cases, we find that the first four PCs are sufficient to explain 88% of the variance in the data (Figure 3, inset). By inspecting the feature decomposition of these four PCs, we found that PC1 (Figure 3A) is homogenously composed of all features, indicating that all extracted features appear to find a linear bisection of the data cloud. The largest contribution was found for amplitude-based features, such as the maximum rms, whereas the modulation index had the smallest weight along this PC. In contrast, PC2, PC3, and PC4 (Figures 3B–D) are clearly dominated by subsets of similar features: features related to the oscillation duration (PC2), features related to the trough interval distributions (PC3), or features related to spectral power (PC4).

**Figure 4:**
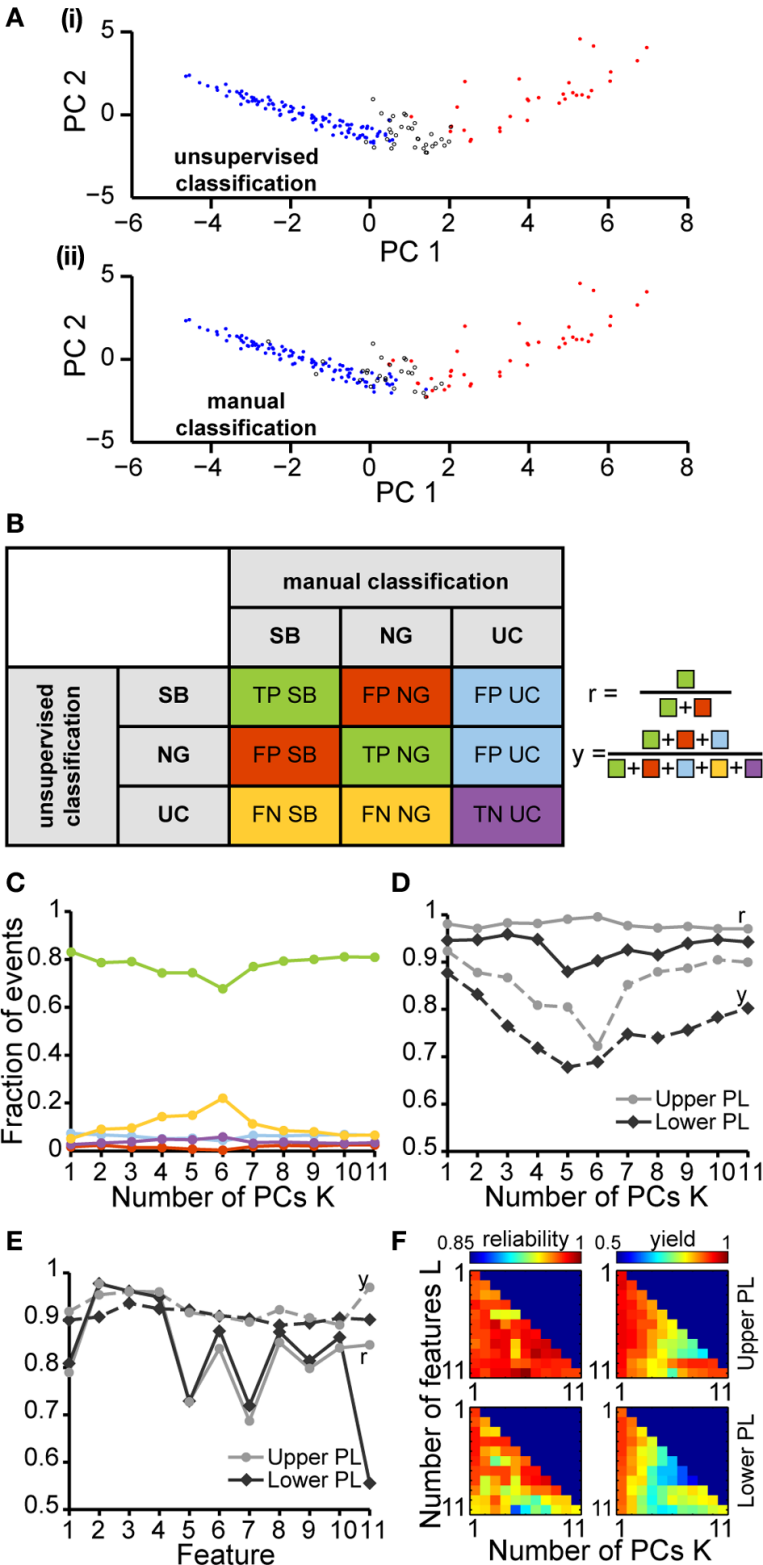
**Unsupervised classification of oscillatory events in the PL of neonatal rats. (A)** Validation of the unsupervised classification **(i)** by comparison with the manual classification **(ii)** of oscillatory events from the same recording. Each dot corresponds to the projection of the feature vector of one event into the principal component space spanned by the first two principal components. The colors code the results of the classification (SB, blue; NG, red). Events that cannot be assigned to one class of oscillations are displayed as white dots (unclassified events, UC). **(B)** Left, table defining the results of unsupervised classification vs. manual classification. Oscillatory events classified as either SB or NG by both methods were defined as true positives (TP_SB_, TP_NG_, green), whereas events wrongly assigned by the unsupervised method when compared to the manual classification were defined as false positive (FP_SB_, FP_NG_, red). Events that were automatically classified as SB or NG, but were left unassigned by the manual classification were defined as false positive unclassified (FP_UC_, blue). Automatically unclassified events were defined as false negatives (FN_SB_, FN_NG_, yellow) if they were manually classified as SB or NG. True negatives (TN_UC_, purple) were left unassigned after manual and automatic classification. Right, formulas for the calculation of the reliability (r) and yield (y) of the unsupervised method. **(C)** Fraction of events in each group [corresponding colors as defined in **(B)**] in relationship to the number *K* of principal components used for the unsupervised classification. **(D)** Reliability (r, solid curves) and yield (y, dashed curves) of the unsupervised method for oscillatory events recorded in the upper (light gray) and lower PL (dark gray) in relationship to *K*. **(E)** Reliability and yield for an unsupervised classification that used only one single feature. Features 1–11 are shown in the same order as in Figure 3. **(F)** Color plots displaying the reliability (left) and yield (right) of the unsupervised classification using different numbers of features (L) and PCs (K) for the upper (top) and lower (bottom) PL. For each row of the matrix, the L features with the highest reliabilities were taken into account. In **(C–E)**, lines connecting data points are for illustrative purposes.

In a second step, we tested how well a partition based on these PCs is able to cluster the SB and NG. To this end, we performed a classification of all six datasets using the unsupervised method on all 11 features. This algorithm was initially based on a cluster analysis in space of the first *K* = 4 PCs that largely explained the variance in the data (Figure 4A). In parallel, we performed a classification of the same data through visual inspection by an experienced researcher. Each event was either labeled as SB, NG or was left UC in case no clear decision could be made by eye. Likewise, each event was identified as SB, NG, or UC based on the classification scheme described above (UC indicated events that did not fall into the SB or NG clusters based on a threshold of 0.7). Plotting the classification results in feature space led to a strong overlap between the automated segmentation and visual inspection. In order to quantify this similarity, we calculated the number of true positives (TP_SB_ and TP_NG_; correctly identified events), false positives (FP_SB_ and FP_NG_; incorrectly assigned events), false positive unclassified (FP_UC_; events that were manually marked as unclassified), false negatives (FN_SB_ and FN_NG_; events manually but not automatically classified), and true negative unclassified (TN_UC_; events unclassified by both methods) (Figure 4B). We observed that the number of TP events depended on the number *K* of PCs used in the classification process (Figure 4C). In particular, as *K* increased starting from *K* = 1 the decrease in the fraction of true positives (from 83.3% at *K* = 1 to 67.7% at *K* = 6) was largely compensated by an increase in the fraction of UC events (from 5.1% at *K* = 1 to 22% at *K* = 6).

To quantify the results of the unsupervised classification, we defined two performance indices: (i) the reliability r = (TP_SB_+TP_NG_)/(TP_SB_+TP_NG_+FP_SB_+FP_NG_), which mirrored the output match of manual and unsupervised classification (ignoring UC events), and (ii) the yield y = (TP_SB_+TP_NG_+FP_SB_+FP_NG_+FP_UC_)/(total number of events), which reflected the fraction of events that were automatically assigned. Consistent with the observations reported above, for both upper and lower PL the reliability was high and independent of K. In contrast, the yield dropped as more PCs were used in the analysis, indicating an increasingly conservative classification (Figure 4D). Therefore, the proposed method reliably distinguished between SB and NG.

Since the unsupervised categorization was possible based on a linear classification using only PC1, we next assessed whether considering a single feature would lead to similar results. For this, we individually quantified the reliability r and the yield y of discrimination for each of the 11 features using a feature vector of length 1 (Figure 4E). While the yield was rather constant across the features, some features, such as the maximum rms, were able to distinguish SB and NG with high accuracy, whereas other features, such as the flatness or number of cycles had poor performance on their own. The most reliable features were: maximum rms, maximum negative peak, maximum slope, and the number of cycles. The performance of the modulation index strongly differed between the upper and lower layers of the PL, since the HFOs are typically more pronounced in the upper layers.

To investigate the reliability and yield as a function of both the number of features and the number of PCs, we performed the classification by varying the number *L* of features with the highest reliabilities (as resulting from Figure 4E), and the number *K* ≤ *L* of PCs. We found that in order to maximize the yield of the classification for neonatal events, only the first PC (i.e., values for *K* = 1) should be used for clustering (Figure 4F, right graphs). If more principle components are considered (*K* > 1), the yield decreased. Therefore, the classification procedure is explained by linear classifiers, suggesting that thresholds placed on the individual distributions are sufficient. Moreover, for the neonatal data the classification performance was high and showed little, if any, dependence on the number of features considered (Figure 4F, left graphs, matrix column *K* = 1 selected according to maximum yield). Even a clustering based on the single most effective feature (i.e., the maximum rms) led to consistently high reliability and high yield (Figure 4F, *L* = 1 and *K* = 1). This is mainly due to the large amplitude of neonatal NG, as expressed in the distributions of the maximum rms (Figures 2B, 4E).

## Results

### Estimation of the classification reliability and performance for oscillatory events from pre-juvenile rats

As described above we firstly developed and validated the unsupervised method for detection and classification of discontinuous patterns of oscillatory activity on prelimbic SB and NG from neonatal (P7–9) rats. We showed that a highly reliable classification of these events is obtained even when using single or few features only.

**Figure 5:**
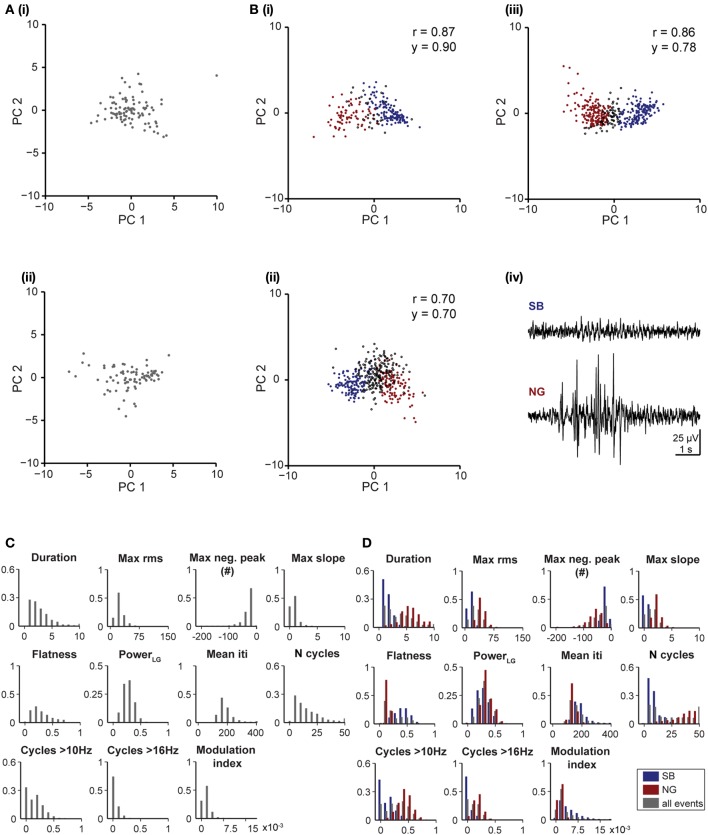
**Unsupervised classification of oscillatory events in rat V1 and mouse PL. (A)** Clustering of oscillatory events recorded in V1 of two P6 rats. Each dot corresponds to the projection of the feature vector of one event into the principal component space spanned by the first two principal components. **(B)** Unsupervised classification of oscillatory events (SB in blue, NG in red, UC in white) recorded in the PL of two P8 **(i,ii)** and one P10 **(iii)** mouse. **(iv)** LFP traces displaying examples of oscillatory events assigned by the unsupervised method as SB or NG. The signals were filtered between 4 and 100 Hz. **(C)** Histograms displaying the distribution of values for distinct features of oscillatory events in the V1 region of neonatal rats. **(D)** Histograms displaying the distribution of values for distinct features of SB (blue bars) and NG (red bars) and for all events (gray bars) in the PL of neonatal mice.

To decide whether the developed unsupervised algorithm is specie- and pattern-independent, we tested its performance on LFP recordings from the V1 of neonatal rats and the PFC of neonatal mice. In line with previous findings (Hanganu et al., 2006), the algorithm identified one pattern of activity in the V1 of neonatal rats, as shown by the single data cloud into principal component space (PC1, PC2) (Figure 5A) and the unimodal distribution of feature histograms (Figure 5C). In contrast, two patterns of activity that correspond to SB and NG have been classified in the PFC of neonatal mice (Figure 5B) and confirmed by the calculated reliability and yield as well as by the bimodal distribution observed in all feature histograms (Figure 5D).

**Figure 6:**
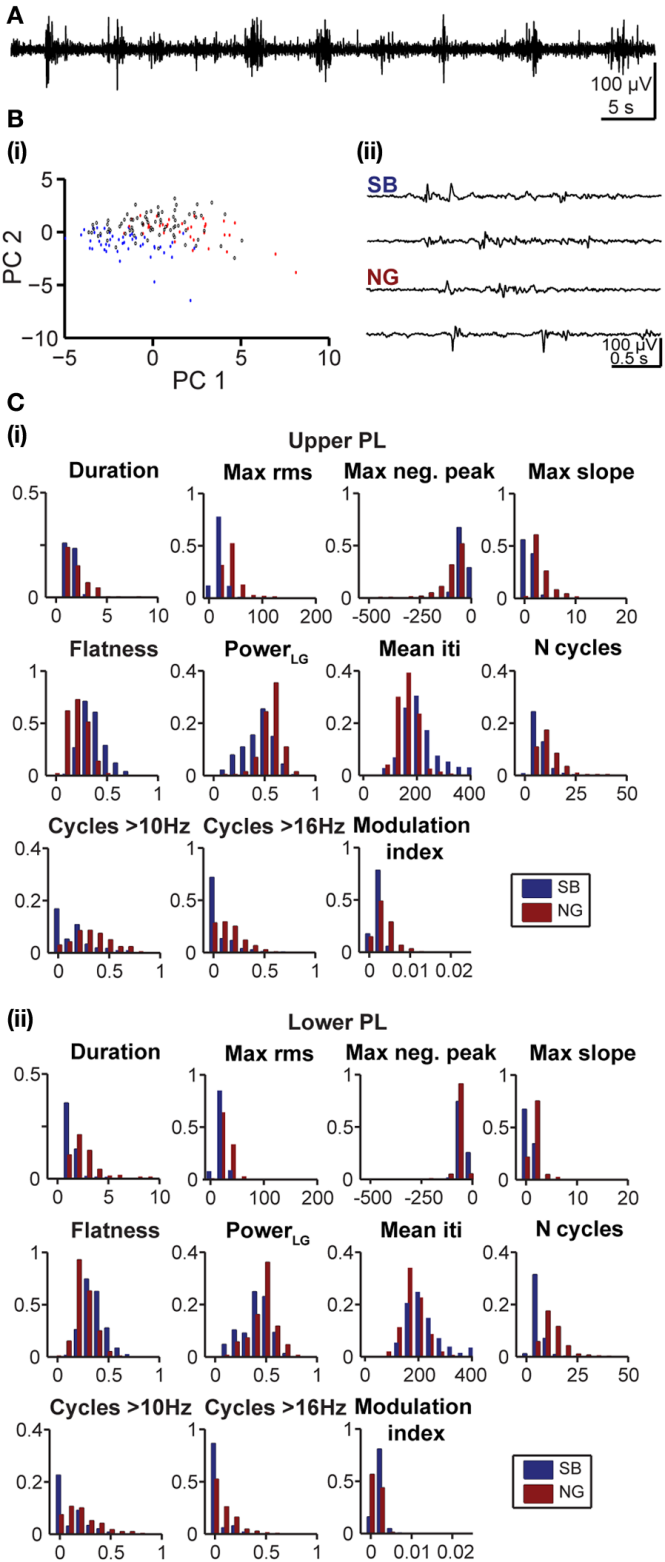
**Feature distribution of oscillatory events in the PL of pre-juvenile rats. (A)** Extracellular LFP recording (4–100 Hz) of the prefrontal oscillatory activity during the developmental switch from discontinuous to continuous pattern (P12 rat). **(Bi)** Automatic classification of oscillatory events in the PL of a P12 rat. Each dot corresponds to the projection of the feature vector of one event (SB in blue, NG in red, UC in white) into principal component space spanned by the first two principal components (PC1, PC2). **(ii)** LFP traces displaying examples of oscillatory events assigned by the unsupervised method as SB or NG. Note the similar appearance of the events that hampers their classification upon visual inspection. The signals were filtered between 4 and 100 Hz. **(C)** Histograms displaying the distribution of values for distinct features of SB (blue bars) and NG (red bars) in the upper **(i)** and lower **(ii)** PL of pre-juvenile rats.

**Figure 7:**
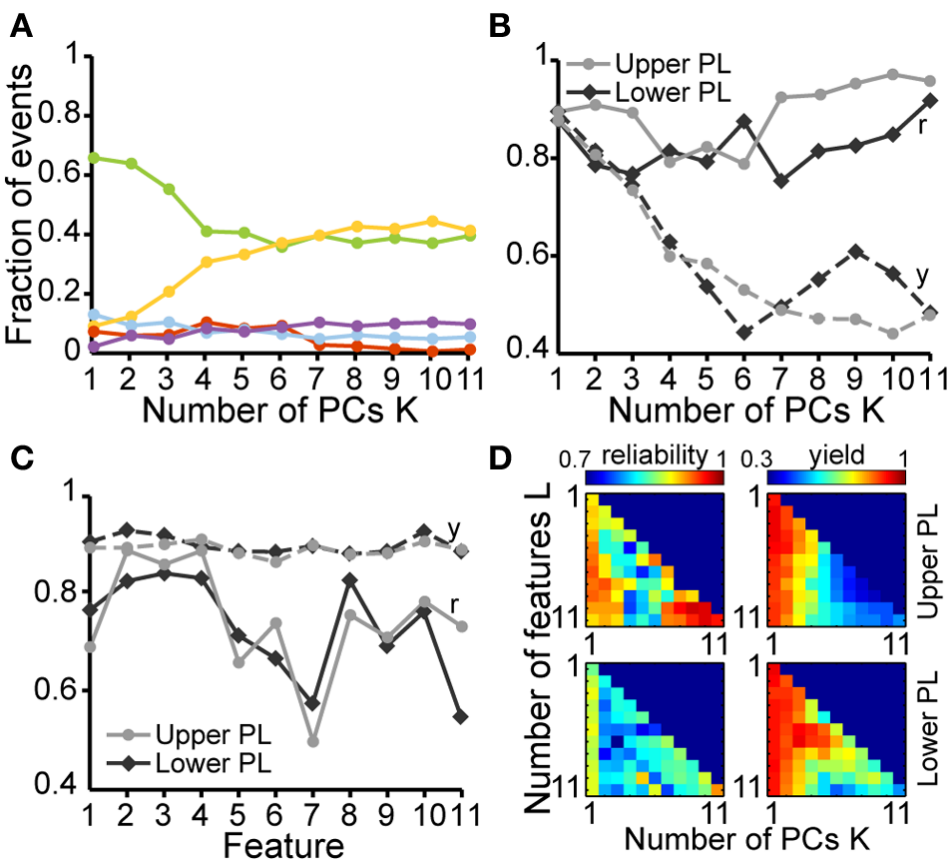
**Classification performance of the unsupervised method applied on the oscillatory events of pre-juvenile rats. (A)** Fraction of TP (green), FP (red), and FN (yellow), SB and NG as well as FP (blue), and TN (purple) UC for different numbers of PCs used for the unsupervised classification. **(B)** Reliability (r, solid curves) and yield (y, dashed curves) of the unsupervised method in relationship to the number *K* of used PCs for the upper (light gray) and lower PL (dark gray). **(C)** Reliability and yield for an unsupervised classification that used only single features. Features 1–11 are shown in the same order as in Figure 3. **(D)** Color plots displaying the reliability (left) and yield (right) of the unsupervised classification using different numbers of features (L) and PCs (K) for the upper (top) and lower (bottom) PL. For each row of the matrix, the same L features as in Figure 4F were taken into account. In **(B,C)**, lines connecting the data points are for illustrative purposes.

At neonatal age the oscillatory events are easily distinguishable and, despite a high degree of inter-event variability, tend to exhibit clear features. In contrast, as the activity starts to switch toward continuous oscillatory rhythms at pre-juvenile age, SB and NG tend to become less obvious in their features and the classification becomes increasingly difficult (Figure 6A). To investigate the applicability of the developed method under these conditions, we further analyzed its performance as a function of age and cortical location. In pre-juvenile animals (P10–12) the data clouds were much less separable in the feature space when compared to neonatal clusters and the unsupervised algorithm produced a larger number of UC events (Figure 6Bi). The oscillatory patterns at this age exhibited less clear features and were difficult to distinguish by visual inspection (Figure 6Bii). Even the most effective features for classification of neonatal SB and NG, such as the max rms, showed no clear differences in their distribution between events (Figure 6C). Nevertheless, despite this high variability, the fuzzy nature of the clustering approach allowed to indubitably identify SB and NG. However, the number of true positive results yielded from the comparison of the unsupervised vs. manual classification was, as expected, lower for the pre-juvenile than for neonatal oscillatory events. The fraction of automatically UC events increased and consequently, the fraction of true positive results decreased with the number *K* of PCs used for classification (Figure 7A). The yield of the classification decreased with *K* for the upper and lower layers of the PL. However, the highest reliability of the method was obtained when all PCs (*K* = 11) were used (Figure 7B). While the reliability of classification obtained by the use of single features is highly dependent on the choice of the feature for both neonatal and pre-juvenile events, it is generally lower for the latter (Figure 7C). We investigated the optimal reliability and yield as a function of both *L* and *K* (Figure 7D). As found for neonatal data, the highest yield is observed for classification in one PC dimension (Figure 7D, right graphs, matrix column at *K* = 1). However, in contrast to the neonatal data, for all prelimbic layers the use of multiple features (*L* > 1) led to a higher reliability (Figure 7D, left graphs, matrix column *K* = 1 selected according to maximum yield) than using the best single feature alone (*L* = 1, *K* = 1). A good yield and the highest reliability for pre-juvenile events was obtained for *L* = 7 (reliability: 0.93) in upper, and *L* = 3 (reliability: 0.88) in lower PL. The reliability of the unsupervised method of classification can be further increased when taking all features (*L* = 11) and all PCs (*K* = 11) into account (Figure 7D, left graphs), but due to the large number of UC events, the yield will be very low (Figure 7D, right graphs). In summary, for difficult data sets, such as the pre-juvenile data presented here, the linear clustering based on the first principal component is still the optimal method. The combination of multiple features provides an advantage over the classification based on a single feature.

### Assessment of spatial and temporal synchrony over the developing prelimbic cortex using automatically classified oscillatory events

**Figure 8:**
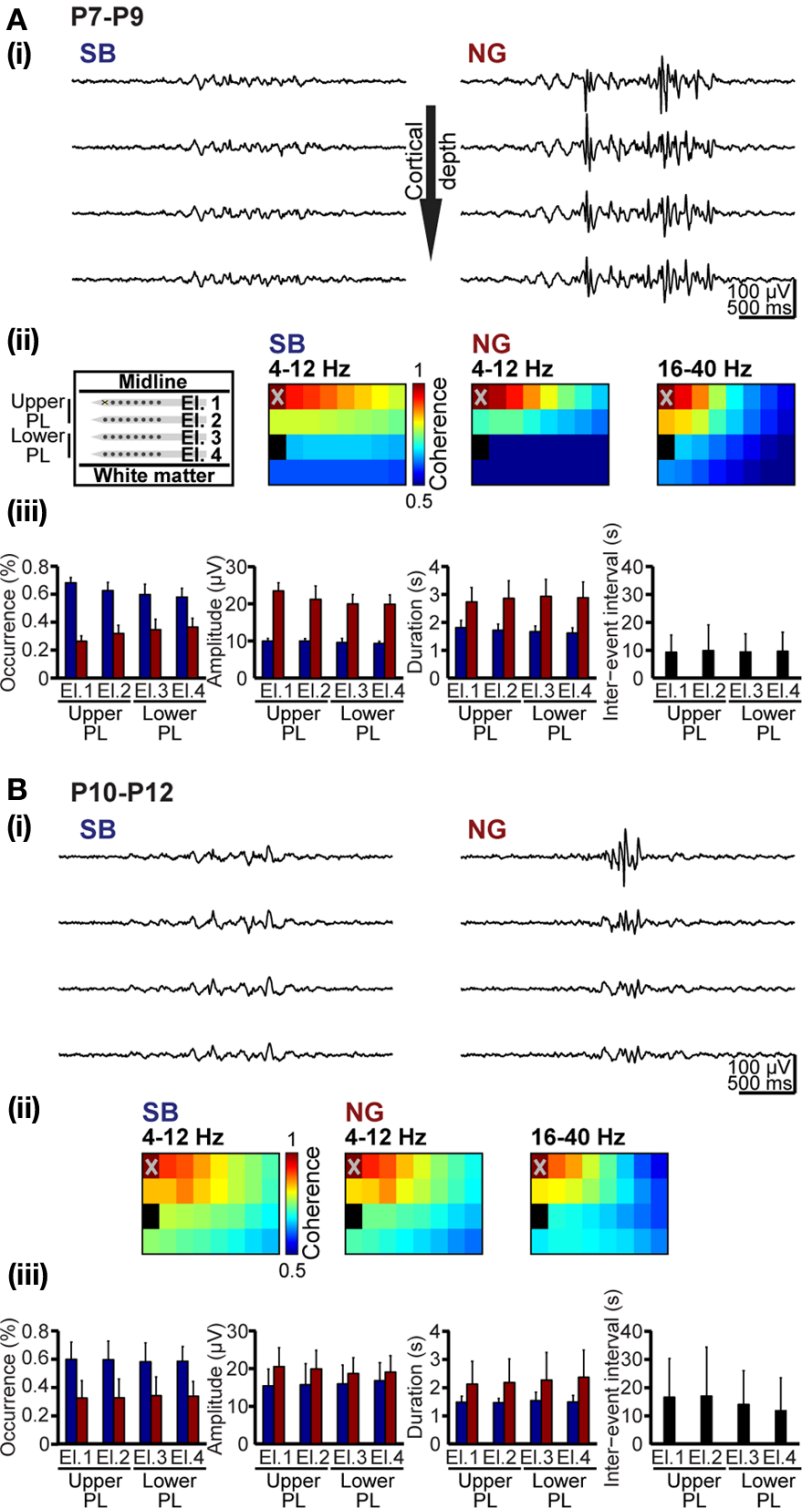
**Spatial and temporal organization of synchrony patterns over the developing PL. (A)** Frequency-dependent synchrony over the upper and lowers layers of the PL in neonatal rats. **(i)** Characteristic SB (left) and NG (right) recorded at different cortical depth (corresponding to El.1–4) and displayed after band-pass filtering (4–100 Hz). **(ii)** Color-coded mean coherence coefficients maps calculated for the frequency components 4–12 Hz of SB and NG as well as for 16–40 Hz of NG recorded from 6 P7-9 rats with 4 × 8 arrays (left panel). The deepest recording site on the electrode shank closest to the midline (marked by X on El.1) was chosen as reference channel. **(iii)** Properties of SB and NG detected and classified using the unsupervised method. Bar diagrams display the occurrence, amplitude and duration of neonatal SB (blue bars) and NG (red bars) as well as duration of inter-event intervals on El.1–4. **(B)** Frequency-dependent synchrony over prelimbic layers of pre-juvenile rats. **(i)** Characteristic SB (left) and NG (right) recorded at different cortical depth (corresponding to El.1–4) and displayed after band-pass filtering (4–100 Hz). **(ii)** Color-coded mean coherence coefficients maps calculated for the frequency components 4–12 Hz of SB and NG as well as for 16–40 Hz of NG recorded from 6 P10-12 rats with 4 × 8 arrays (left panel). The deepest recording site on the electrode shank closest to the midline (marked by X on El.1) was chosen as reference channel. **(iii)** Properties of SB and NG detected and classified using the unsupervised method. Bar diagrams display the occurrence, amplitude and duration of pre-juvenile SB (blue bars) and NG (red bars) as well as duration of inter-event intervals on El.1–4.

Unbiased classification of oscillatory events represents the prerequisite for characterization of their spatial and temporal organization over neocortical areas. We exemplified this application aiming at deciding whether SB and NG differently synchronize the developing PL. Oscillatory events recorded at multiple sites over the cortical depth were classified using the unsupervised method described above (Figures 1A, 8A). In all recordings, both SB and NG were not restricted to one recording site, but occurred simultaneously at several neighboring sites of the 4 × 8 recording array. To estimate the spatiotemporal dimension of frequency-dependent SB- and NG-mediated functional coupling through synchrony, the coherence coefficients for the 4–12 Hz and 16–40 Hz frequency bands of oscillatory events and for all recording sites were calculated and color-coded in relationship to one reference channel marked by X (Figures 8Aii,Bii). Overall, the coherence between neighboring recording sites was rather high and decreased with distance from the reference recording site. However, the rate of decrease depended on the frequency and directionality of selected electrode pairs, i.e., within vs. across layers. Neonatal SB with dominant power within 4–12 Hz exhibited the strongest synchrony within the same layer (coherence decrease 0.4 ± 0.14/mm), whereas the coherence coefficients rapidly decreased across layers (0.67 ± 0.4/mm). Similarly, the synchrony within theta–alpha band of neonatal NG was stronger within (coherence decrease 0.66 ± 0.08/mm) than across layers (coherence decrease 0.92 ± 0.18/mm). In contrast, the 16–40 Hz component of NG was synchronized in a column-like pattern since the coherence decreased stronger within (0.91 ± 0.32/mm) than across layers (0.63 ± 0.16/mm) (Figure 8A). The spatial organization of synchrony changed significantly with age (Figure 8B). The column-like coupling of oscillatory activity in beta-gamma frequency (coherence decrease 0.68 ± 0.13 within layers and 0.54 ± 0.15 across layers) as well as the 4–12 Hz synchrony within layers (coherence decrease 0.45 ± 0.22 for NG and 0.47 ± 0.21 for SB) became less precise in P10–12 rats. Generally, the pre-juvenile period seems to represent a time window of reorganization of oscillatory coupling within the PL, the decrease of coherence coefficients being similar within and across layers for all frequency bands and oscillatory events.

Additionally, the unsupervised method for detection and classification of oscillatory events can be applied for unbiased quantification of the SB and NG properties at neonatal and pre-juvenile age (Figures 8Aiii,Biii). At all investigated developmental stages and over the entire prelimbic depth, the probability of occurrence of SB was higher, but their duration was shorter and their amplitude smaller when compared with the NG. These differences between SB and NG were highly significant (*p* < 0.001). In neonatal rats, the NG were more prominent in the upper layers of the PL and the cortical plate. With ongoing maturation and the onset of switch from discontinuous to continuous oscillatory activity, the differences between SB and NG became less obvious, but were still significant (*p* < 0.001). These results confirm our previous observations (Brockmann et al., 2011), supporting the general applicability of the classification method for unbiased analysis of oscillatory patterns of activity in the developing brain.

## Discussion

In this methodological study we have developed and explored the use of a novel unsupervised algorithm for detecting and classifying the patterns of oscillatory activity in the developing brain. This method involved three steps: the identification of oscillatory events using a threshold procedure, the extraction of up to 11 quantitative features from these events, and an unsupervised clustering of the resulting feature vectors corresponding to the events. We demonstrate that (i) the rms-based method reliably and almost independently of signal-to-noise ratio detects the periods with network oscillations; (ii) the discontinuous cortical activity of neonatal and pre-juvenile rodents can be characterized by 11 features that we defined on a per-event basis; (iii) due to the high degree of variability between events of the same type, the heuristically determined features differ in their ability to classify the oscillatory events (best features: maximum rms, maximum negative peak, maximum slope, number of cycles and modulation index); (iv) while for clearly distinguishable oscillatory events single features (e.g., amplitude-based) may be sufficient for a powerful linear classification, the inclusion of a large number of features improves the reliability of the algorithm without loss of the yield for manually difficult to detect and classify pre-juvenile oscillatory events. Finally, we demonstrated how the unsupervised characterization of discontinuous oscillatory patterns opens new perspectives for unbiased analysis of spatial organization in the developing brain.

Reliable detection and classification of SB and NG despite their inter-event variability represent the pre-requisite for assessing the spatial and temporal dynamics of early oscillatory activity in the PL. In this study, we implemented a method to automatically identify the two types of events using a PCA with subsequent unsupervised clustering. The method was shown to classify events at comparable precision as human observers across a number of different recording sessions without the need for session-specific parameter adjustments, e.g., a threshold on individual features. In addition to providing an objective measure to distinguish SB from NG, the PCA allowed to identify features of the events that best highlight their differences. The results revealed two characteristics that mainly differ between SB and NG. First, large maximal values for the rms are indicative of NG. These events tended to show rapid, short and large deflections of the amplitude, in particular during the start of the event. The maximum negative peak and the maximum slope of the rms were further, yet less characteristic amplitude-dependent signatures for NGs. Second, an increase in the number of cycles represented a powerful feature for the detection, as NGs typically exhibited a composition of not only slow (4–12 Hz), but also fast (16–40 Hz) oscillatory periods.

Feature vectors were clustered using a fuzzy clustering algorithm. Instead of assigning each event exclusively to one of the two postulated *a priori* clusters (SB and NG), it assigns a membership degree to each of the clusters. Introducing a threshold (i.e., events which fail to exceed the minimum membership degree for any of the two clusters are discarded), the algorithm flexibly allowed to adjust the sensitivity of the analysis by regulating the number of UC events. We point out that the notion of yield introduced here is a direct consequence of the fuzzy property of the clustering algorithm, reflecting the percentage of UC events. In this way, it enabled reliable identification of SB and NG even for recordings that exhibit different signal-to-noise ratios or display a large inter-subject variability. Our preliminary analysis revealed that the fuzzy clustering algorithm is not the unique method of detection and classification of oscillatory events. In particular, we showed that the optimal classification is obtained by using the first principal component, suggesting a linear classification. Similar clustering of SB and NG could be achieved using standard unsupervised clustering algorithms, such as k-means clustering, albeit at lower performance (Figure S2 vs. Figure 4D) as the inability of k-means algorithm to leave events UC leads to more incorrectly classified events.

For the practical implementation of the method the following two considerations appear as beneficial. First, multiple features should be used to construct the feature vectors. While for some data sets, even single features may already yield a good classification performance, the use of multiple features was shown to boost reliability, in particular for less well distinguishable data. The features identified to perform with highest performance are (starting from the best): maximum rms, maximum negative peak, maximum slope, number of cycles, number of cycles above 16 Hz, and Power LG. For recordings in upper PL, the modulation index was also a good classification measure. Second, the fuzzy clustering should be used as an approach to define UC events. This strategy allowed for a conservative identification of events by ignoring events that are too far apart from cluster centers in the classification space. It corresponds to a strategy in manual classification, where certain events are ignored if their type cannot be determined.

The relevance of an unsupervised and experimenter-unbiased algorithm is highlighted by the literature findings of the last decades. Extensive investigation of brain rhythms either by invasive (e.g., LFP) or non-invasive (e.g., EEG) methods identified several patterns of network oscillations, the properties of which significantly varied among reports. Their nomenclature is not less heterogeneous. In human preterm babies the multi-band discontinuous events including a low intrinsic frequency nested with fast activity have been termed according to their frequency (e.g., delta waves) or as spontaneous activity transients (Kostovic and Judas, 2002; Vanhatalo and Kaila, 2006; Vecchierini et al., 2007). In contrast, their correspondents in rodents have been classified as early network oscillations (ENOs), SB, nested gamma spindle bursts (NG), long beta oscillations or giant depolarizing potentials (GDPs), to mention the most commonly used terms (Khazipov et al., 2004; Adelsberger et al., 2005; Hanganu et al., 2006; Yang et al., 2009; Brockmann et al., 2011). While the fragmented structure of the events was a common feature of all these patterns, their amplitude, duration, occurrence and frequency distribution significantly varied. In the absence of a unitary and unsupervised method for detection and classification, the differences uncovered by manual methods (i.e., visual analysis) might be not solely caused by the anatomical structure, electrode configuration, presence or level of anesthesia and age, but equally by the individual expertise and settings. The development of the present clustering algorithm offers the possibility to test the existence and source of variability for different patterns of activity in the immature brain. By including a large number of event features the algorithm has not only a high degree of reliability but it is also very flexible and applicable to different experimental conditions (specie, age, brain area). For example, our results demonstrated the ability of clustering algorithm to characterize the oscillatory patterns during the pre-juvenile period of switch from discontinuous to continuous activity. The similar task when achieved manually requires a long-lasting expertise of the analyst. Moreover, our data confirmed the applicability of the algorithm to LFP recordings from neonatal and pre-juvenile mice, the signal-to-noise ratio of which is less prominent and for which the manual detection frequently failed.

However, the present algorithm is based on a phenomenological procedure in the absence of knowledge regarding the underlying cause for different types of oscillatory episodes. We believe that this approach and the features may constitute a promising starting point for fine-tuning of the classification parameters. Nevertheless, even for the data presented in this study, our approach is not parameter-free. While the method allowed for a robust detection based on features that were easily extracted with a very low number of parameters, the details of the detection still depended on the precise choice of parameters and employed features. A certain fraction of false positives and false negatives compared to the ground truth identity of the events is unavoidable. Therefore, in practice one needs to adjust the sensitivity of the detection with respect to the analysis performed on the detected events. For example, if interested in estimating the absolute occurrence of a certain event type, a high classification yield is likely to be preferable to reduce the number of undetected events. In other cases the goal might be obtaining only a few events that clearly belong to the SB or NG category. For this, a low yield is desirable. A similar problem is faced when performing spike sorting, where data variability still prohibits the use a fully automatized, parameter-free procedure, and likewise suggests a close harmonization of sorting strategy and analysis (Pazienti and Grün, 2006).

Additionally, our results revealed that the automatically detected and classified patterns of oscillatory activity may be further used for synchrony analysis over distinct cortical areas at neonatal and pre-juvenile age. The SB with main frequency within theta-alpha band as well as the NG with nested gamma activity on the 4–12 Hz rhythm showed distinct properties in the upper vs. lower layers of the PL. Surprisingly, the coupling by synchrony of the theta–alpha and gamma rhythms of SB and NG was different and changed with age. While the coherence in 4–12 Hz was higher within than between layers, a column-like synchrony entrained the PL in gamma frequency band. In line with the increasing similarity between SB and NG, these coupling differences between low and high frequency activity diminished with age. The column-like synchrony is present in the pre-juvenile PL, although its pattern appears less precise than at neonatal age. This might be due to the reorganization of circuitry and pruning of connections (Changeux and Danchin, 1976; Zuo et al., 2005), peaking at the age of switch from discontinuous to continuous activity, previously reported to occur at the end of the second postnatal week (Brockmann et al., 2011). The spatial organization of oscillatory events that have been detected and classified by the unsupervised clustering algorithm let us suggest that the rodent PL may be functionally mapped in a column-like pattern. This pattern of functional organization seems to be related to the early gamma entrainment of neonatal networks and to both theta and gamma rhythms at juvenile developmental stage. These findings complement the recently shown contribution of oscillatory synchrony to the functional refinement of the PFC (de Almeida et al., 2013).

## Funding

This work was supported by the Emmy Noether-Program of German Research Foundation (Ha4466/3-1 to Ileana L. Hanganu-Opatz), Priority Program 1665 of the DFG (Ha4466/8-1 to Ileana L. Hanganu-Opatz and Michael Denker), Boehringer Ingelheim Fonds travel grant (to Nicole B. Cichon) and German Federal Ministry of Education and Research (01GQ0809 to Ileana L. Hanganu-Opatz).

### Conflict of interest statement

The authors declare that the research was conducted in the absence of any commercial or financial relationships that could be construed as a potential conflict of interest.

## Footnotes

^1^chronux.org. Available online at: http://chronux.org/. (Accessed November 5, 2012).

## Supplementary material

Figure S1 **Detection of oscillatory events**. Histograms of root-mean-squared (rms) amplitude (gray) of the recorded LFP from a P8 **(A)** and P12 **(B)** rat. Black curves display the Gaussian functions fitted to the left part of the rms histogram (values from 0 to the histogram peak) which corresponds to the time-windows of suppressed oscillatory activity.

Figure S2 **Unsupervised classification using k-means clustering**. (A) Classification of oscillatory events from the same data set as shown in Figure 4A. Each dot corresponds to the projection of the feature vector of one event (SB in blue, NG in red) into principal component space spanned by the first two principal components. **(B)** Reliability of the k-means classification in relationship to the number *K* of used PCs for the oscillatory events in the upper (light gray) and lower PL (dark gray) of neonatal rats (*n* = 6).
