# Supplementary figures and images for: Unsupervised classification of neocortical activity patterns in neonatal and pre-juvenile rodents
Source: Front Neural Circuits. 2014 May 27;8:50. doi: 10.3389/fncir.2014.00050 (PMC4034041; doi:10.3389/fncir.2014.00050)

**A****P8**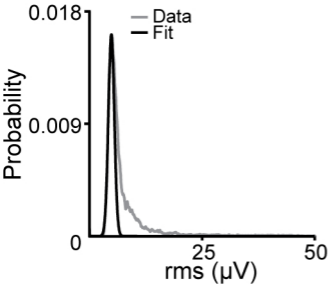**B****P12**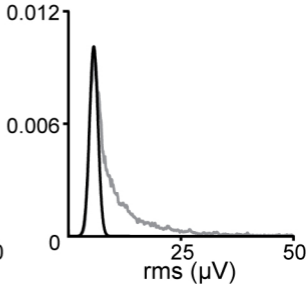

**Cichon et al. - Figure S1**

Supplement: Figure S1 — Detection of oscillatory events. Histograms of root-mean-squared (rms) amplitude (gray) of the recorded LFP from a P8 (A) and P12 (B) rat. Black curves display the Gaussian functions fitted to the left part of the rms histogram (values from 0 to the histogram peak) which corresponds to the time-windows of suppressed oscillatory activity. [file Presentation1.PDF]

**A**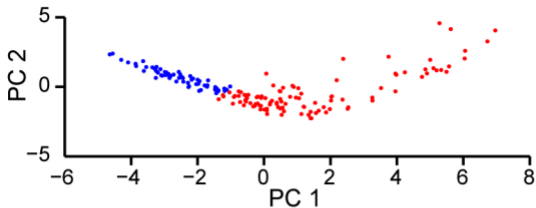**B**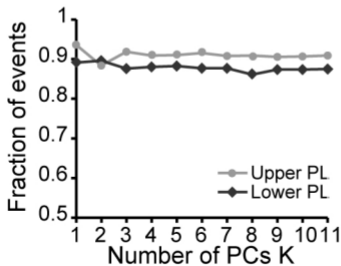**Cichon et al. - Figure S2**

Supplement: Figure S2 — Unsupervised classification using k-means clustering. (A) Classification of oscillatory events from the same data set as shown in Figure 4A. Each dot corresponds to the projection of the feature vector of one event (SB in blue, NG in red) into principal component space spanned by the first two principal components. (B) Reliability of the k-means classification in relationship to the number K of used PCs for the oscillatory events in the upper (light gray) and lower PL (dark gray) of neonatal rats (n = 6). [file Presentation2.PDF]
